# Automated segmentation of craniopharyngioma on MR images using U-Net-based deep convolutional neural network

**DOI:** 10.1007/s00330-022-09216-1

**Published:** 2022-11-18

**Authors:** Chaoyue Chen, Ting Zhang, Yuen Teng, Yijie Yu, Xin Shu, Lei Zhang, Fumin Zhao, Jianguo Xu

**Affiliations:** 1 Department of Neurosurgery, Sichuan University, West China Hospital, No. 37, GuoXue Alley, Chengdu, 610041 People’s Republic of China; 2 Department of Radiology, Sichuan University, West China Hospital, No. 37, GuoXue Alley, Chengdu, 610041 People’s Republic of China; 3 College of Computer Science, Sichuan University, Chengdu, 610065 People’s Republic of China; 4 College of Computer Science, Sichuan University, Chengdu, 610041 People’s Republic of China; 5 Radiology Department, West China Second University Hospital, Sichuan University, No. 20, section 3, Renmin South Road, Wuhou District, Chengdu, 610041 People’s Republic of China

**Keywords:** Craniopharyngioma, Magnetic resonance imaging, Deep learning, Computer-assisted diagnosis

## Abstract

**Objectives:**

To develop a U-Net-based deep learning model for automated segmentation of craniopharyngioma.

**Methods:**

A total number of 264 patients diagnosed with craniopharyngiomas were included in this research. Pre-treatment MRIs were collected, annotated, and used as ground truth to learn and evaluate the deep learning model. Thirty-eight patients from another institution were used for independently external testing. The proposed segmentation model was constructed based on a U-Net architecture. Dice similarity coefficients (DSCs), Hausdorff distance of 95% percentile (95HD), Jaccard value, true positive rate (TPR), and false positive rate (FPR) of each case were calculated. One-way ANOVA analysis was used to investigate if the model performance was associated with the radiological characteristics of tumors.

**Results:**

The proposed model showed a good performance in segmentation with average DSCs of 0.840, Jaccard of 0.734, TPR of 0.820, FPR of 0.000, and 95HD of 3.669 mm. It performed feasibly in the independent external test set, with average DSCs of 0.816, Jaccard of 0.704, TPR of 0.765, FPR of 0.000, and 95HD of 4.201 mm. Also, one-way ANOVA suggested the performance was not statistically associated with radiological characteristics, including predominantly composition (*p* = 0.370), lobulated shape (*p* = 0.353), compressed or enclosed ICA (*p* = 0.809), and cavernous sinus invasion (*p* = 0.283).

**Conclusions:**

The proposed deep learning model shows promising results for the automated segmentation of craniopharyngioma.

**Key Points:**

*• The segmentation model based on U-Net showed good performance in segmentation of craniopharyngioma.*

*• The proposed model showed good performance regardless of the radiological characteristics of craniopharyngioma.*

*• The model achieved feasibility in the independent external dataset obtained from another center.*

**Supplementary Information:**

The online version contains supplementary material available at 10.1007/s00330-022-09216-1.

## Introduction

Craniopharyngioma is a type of uncommon benign intracranial tumors with two histological subtypes called adamantinomatous craniopharyngioma (ACP) and squamous papillary craniopharyngioma (PCP) [1, 2]. As its locally aggressive growth pattern and anatomic location proximal to vital brain structures, it is so-called behavior malignant tumor leading to devastating neurological dysfunction along with significant mortality and morbidity [3, 4]. Patients with craniopharyngioma may suffer from severe symptoms, including endocrine deficiencies, visual impairment, and increased intracranial pressure [5, 6].

Magnetic resonance imaging (MRI) before and after gadolinium injection is now the golden standard imaging for both detection and assessment of craniopharyngioma [7]. The importance of reliable tumor segmentation on MRIs has been highlighted. On the one hand, it can provide a more individual pre-treatment assessment to formulate therapeutic schemes for both neuro-radiologists and neurosurgeons, as segmentation-based three-dimensional assessment was introduced, providing a more sensitive tumor detection and progression monitoring in image reading [8, 9]. On the other hand, in previous researches, a series of segmentation-required models were proposed, and represented feasible ability in pathological discrimination, differential diagnosis, and brain-invasion prediction [10–15]. However, manual delineation is tedious, laborious, and time-consuming, commonly leading to inter-and intra-rater variations that may lead to final analysis deviation [16]. It is urgent to develop a new reliable model to automatically segment craniopharyngioma on routine MRIs.

Deep neural network (DNN) is a multidisciplinary and interdisciplinary research that can shorten the image processing time and improve the reliability of diagnostic results leveraging big data in the field of medical imaging [17, 18]. It has been used as a necessary step in image pre-processing, allowing for a more tumor-specific analysis, whereas comparing to other types of brain tumor, segmentation of craniopharyngioma using DNN is more challenging as its pathologies vary dramatically in their imaging presentation. On routine MRIs, PCP usually presents a noncalcified and solid entity, while imaging features of ACP show a typical combination called 90% rule, that 90% show more or less prominent calcifications, 90% of tumors are predominantly cystic, and about 90% take up contrast in the cyst walls [19, 20]. The variety of imaging presentation hampers the high performance of DNN models in segmentation. In the previous research, classic DNN showed limited performance, and suggested that network should be improved according to the imaging features of craniopharyngioma [14].

Given the importance of tumor segmentation in both clinical routine practice and academic researches, in this study, we aim to develop a novel deep learning model to automatically segment craniopharyngioma on MR images by using convolutional neural networks (CNNs) technology.

## Method

### Patient selection

**Fig. 1:**
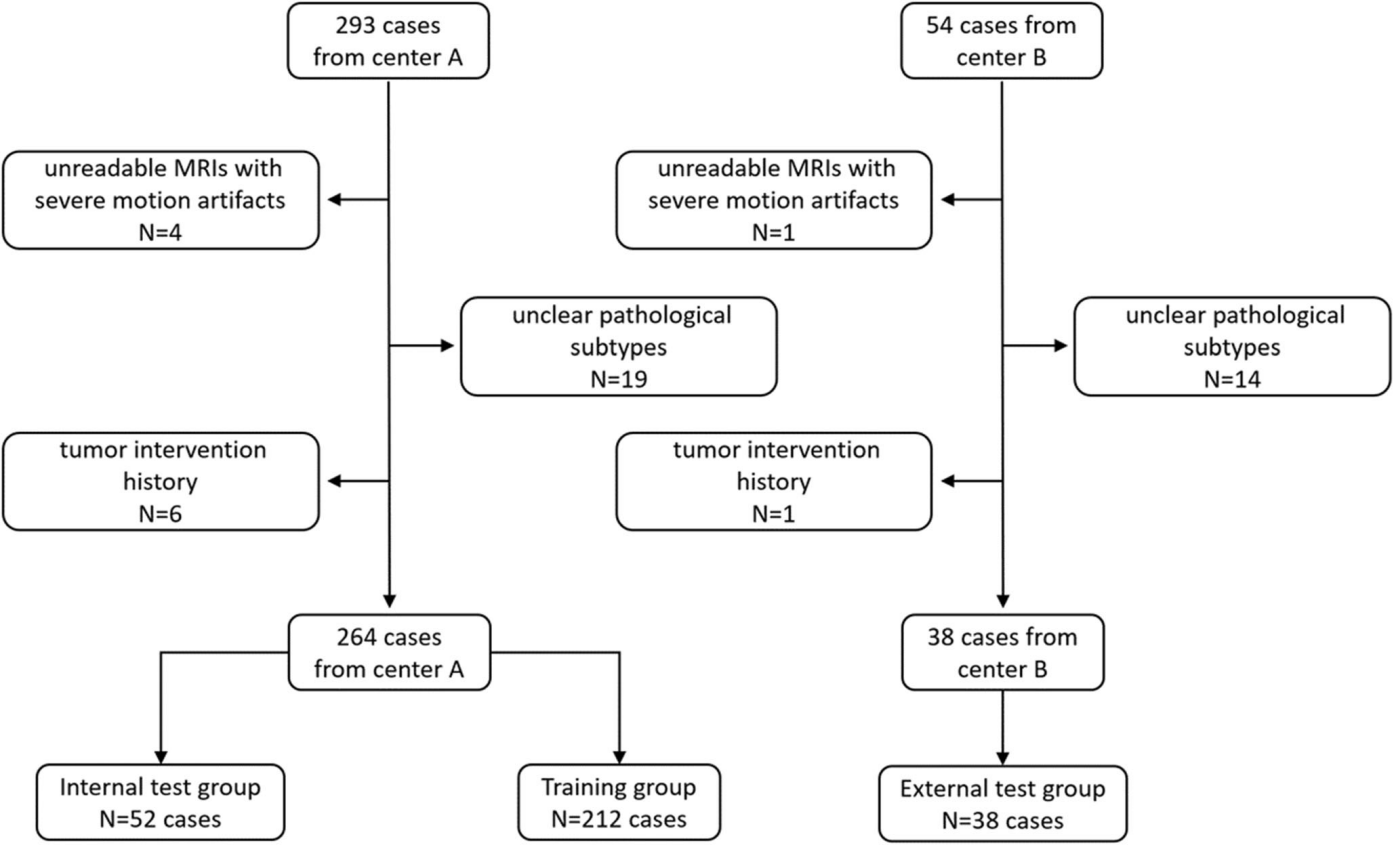
Flowchart of the study population

This was a retrospective study performed in two medical centers. Institutional Review Boards of our hospitals approved our research, and patient consent was waived (2021-S-851). From January 2016 to December 2021, 293 cases and 54 cases with pathologically diagnostic craniopharyngioma were retrieved from medical center A and medical center B, respectively. Patients underwent pre-surgical MR scans in radiology department and received surgical resection in neurosurgery department in our institutions. The exclusion criteria were as follows: (1) unreadable MRIs with severe motion artifacts; (2) tumor intervention history prior to MR scans, such as biopsy and radiotherapy; (3) unclear pathological subtypes. With above inclusion and exclusion criteria, 264 patients from center A and 38 patients from center B were included in the current study. Figure 1 showed the flowchart of involving the study population. The MRIs were exported from the Picture Archiving and Communication System. Clinical characteristics were also collected, including age, gender, tumor characteristics, and pathological findings.

### Sequence selection and MRI acquisition

Four sequences were available, including T1-weighted imaging, T2-weighted imaging, contrast-enhanced T1 images (T1CE), and fluid attenuated inversion recovery. Among these images, T1CE was chosen as it was optimal for identifying tumor boundary. Different from previous researches that mostly used axial images, we chose coronal images as they could provide more details about the relationship between tumor and surrounding structures, especially regarding internal carotid artery (ICA) compression and cavernous sinus invasion. MR scans were performed in the head-first supine position. Gadolinium-DTPA (Beijing BeiluPharmaceutical Corp.) was used as the contrast agent (0.1 mmol/kg).

In center A, MR scans were performed with 3 T Philips Achieva scanners (Philips Corp.) equipped with a head or head–neck coil. Parameters were set as follows: slice thickness was 5 mm; repetition time was 142.2 s; echo time was 2.3 s; phase field of view was 73.44%; acquisition matrix was 0/256/204/0; flip angle was 90°; average series time was 27.24 min. In center B, MR scans were performed with 1.5 T Siemens Sonata scanners (Siemens AG Corp.) equipped with a head or head–neck coil. Parameters were set as follows: slice thickness was 4 mm; repetition time was 631s; echo time was 7.7 s; phase field of view was 75%; acquisition matrix was 0/256/144/0; flip angle was 80°; average series time was 29.15 min.

### Image pre-processing and ground truth segmentation

The images were resized to 256 × 256, resampled spatial resolution to 1 × 1 × 1, and normalized to [0,1]. Contrast limited adaptive histogram equalization approach was also used to enhance the images.

The manual segmentation was performed on coronal images by using ITK-SNAP platform (version 3.80) [21]. Two neuroradiologists manually draw the regions of interest (ROIs) along the tumor boundary in consensus reading, excluding peripheral blood vessels (mainly referred to ICA) or invaded structure (mainly referred to cavernous sinus). All contours were reviewed by two senior neuroradiologists (the corresponding authors, with 20 years of experience in image reading). Twenty cases were randomly selected and segmented another time to examine intraclass reproducibility by another radiologist with a 10-day interval.

### U-Net framework architecture and training

**Fig. 2:**
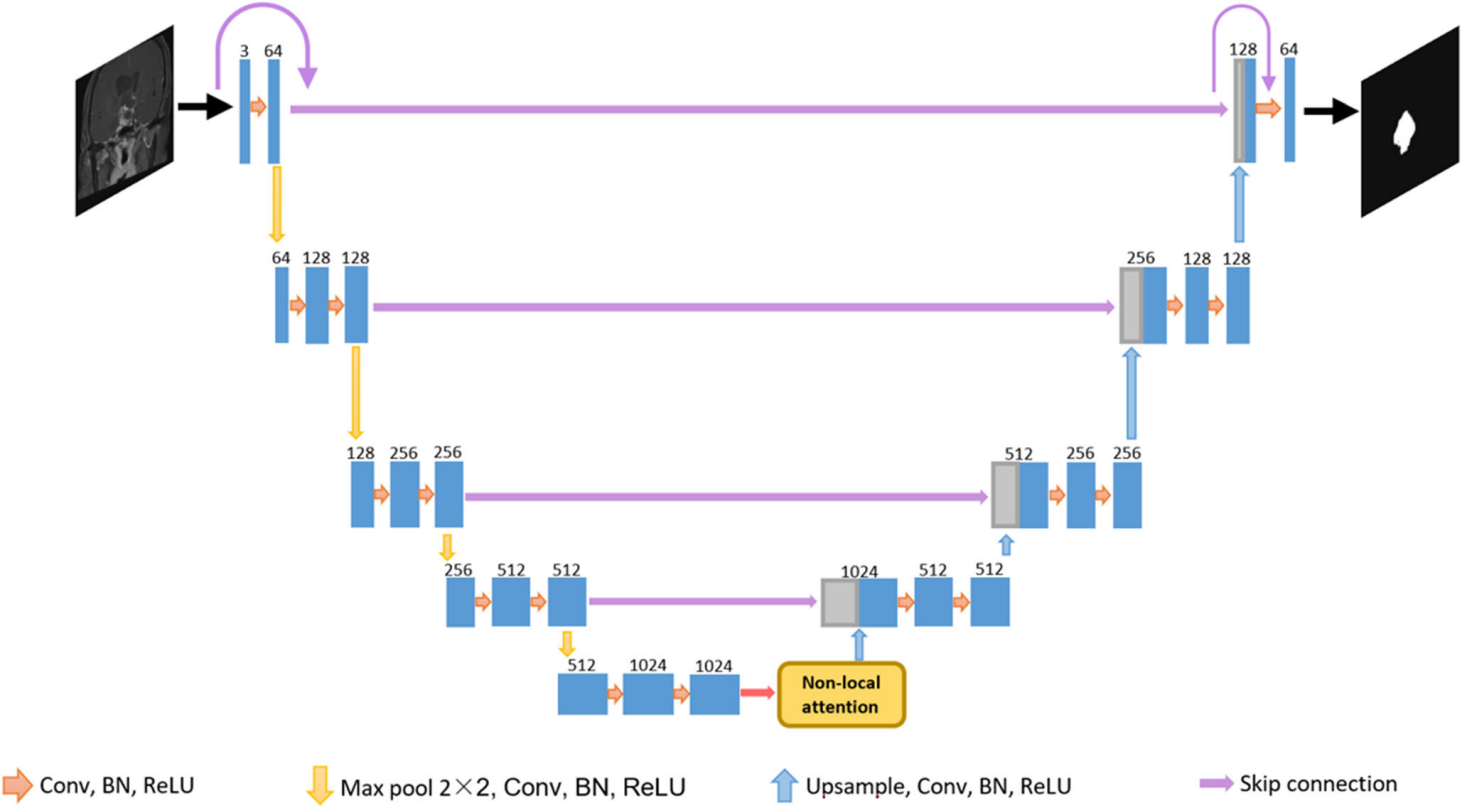
The basic structure of the modified U-Net. A none-local block is added at the bottom layer of the network to capture the long-range information of tumor images

The networks were implemented in Pytorch framework. Data augmentation was also adapted that the images were randomly flipped horizontally and vertically, and rotated within the range of −10 to 10 degree for each epoch. Standard U-Net structure was used for modeling. Based on the model performance and image characteristics of craniopharyngioma, the network was improved from two aspects, that first, a non-local attention block was introduced to capture long-range dependencies in images [22]; and second, the loss function was changed as *loss total = 0.5 × loss Tversky + 0.5 × loss cross entropy* [23, 24]. Hyperparameters of Tversky loss was set as α = 0.1, β = 0.9, respectively. The network structure used in this research is shown in Fig. 2.

**Fig. 3:**
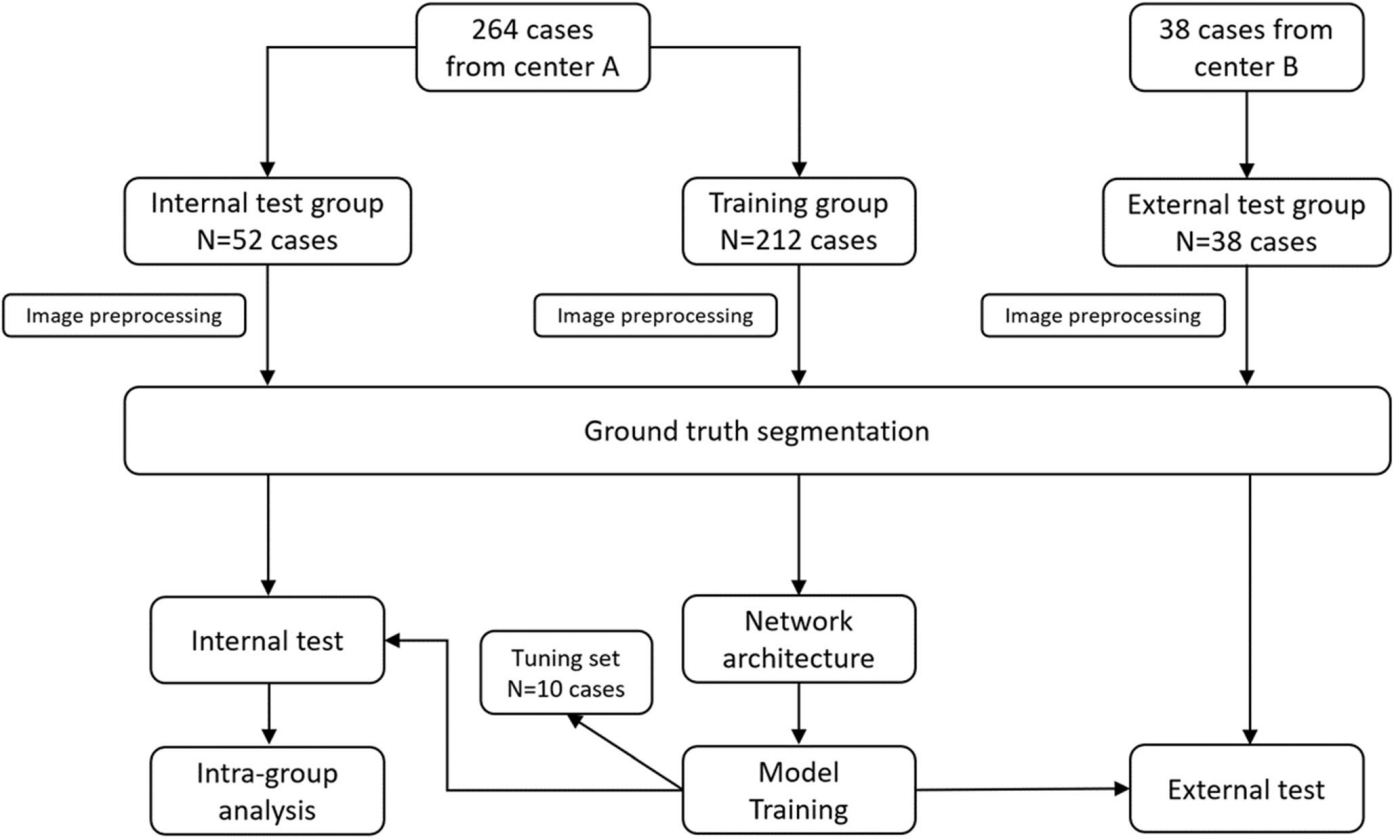
Workflow of the development of the convolutional neural networks CNN models

Figure 3 shows the workflow of the development of the deep CNN model. The cases from center A were randomly divided into the training group (*N* = 212) and the internal test group (*N* = 52) on the proportion of 8:2; cases from center B were used as the independent external test group (*N* = 38) to test model generalization. Ten cases were randomly selected from the training group, and set as tuning set to validate the performance in each training epoch. As for the network parameters, input size of network was set as 256 × 256, and optimizer was set as adam. The batch size of the sections was set to 8, and the learning rate was set to 1e-5. The number of epochs for the model training was set to 300.

### Evaluation metric

The trained model performance was quantitatively evaluated in both internal and external test group by comparing ground truth masks with predictions in pixel level. For each case, dice similarity coefficients (DSCs) were calculated as indicator of similarity by measuring the overlap of segmentations. Hausdorff surface distance (HD) were calculated as indicator of the distance that the contours in predicted segmentations have to move to enclose the contours in manual ROIs. Classic quantitative metrics were also calculated, including Jaccard value, true positive rate (TPR), false positive rate (FPR), tumor volume, and calculation time. The definitions of the metrics were provided in Supplement Material 1.

All statistical analyses were performed by using IBM SPSS Statistics Version 25.0 (IBM Corp.). Continuous variables were summarized as mean value, and categorical variables were summarized by number and percentage. The significance of intra-group difference was evaluated by using one-way ANOVA analysis. Bland-Altman plots and interclass correlation coefficients (ICCs) were used to access the intra-observer reproducibility and the difference between manual segmentation and automatic segmentation.

## Result

### Patient characteristics

A total number of 302 patients were included in this research. For the all patients involved in this research, the mean age was 32.92 years old, and the gender ratio of participants was 179:123. A total number of 230 patients were pathologically diagnosed with ACP, and 72 patients were diagnosed with PCP, respectively. The image characteristics of tumor were variant, that 34.4% were predominantly solid, 17.9% were predominantly cystic, and 47.7% were mixed solid-cystic. Lobulated appearance was found in 43.0% patients. Moreover, compressed or enclosed ICA was found in 40.1% cases, and invaded cavernous sinus was found in 54.0% cases. Table 1 shows the detailed patients’ characteristics and histopathological information.

**Table 1:** Clinical, histopathological, and radiological features of patients

	Center A	Center B	Total	Percentage
Age				
Mean	32.25	37.60	32.92	–
Range	14–58	7–49	7–58	–
Gender				
Male	157	22	179	59.3%
Female	107	16	123	40.7%
Pathological subtype				
ACP	201	29	230	76.2%
PCP	63	9	72	23.8%
Predominantly composition				
Cystic	91	13	104	34.4%
Solid	46	8	54	17.9%
Mixed solid-cystic	127	17	144	47.7%
Lobulated shape	112	18	130	43.0%
Compressed or enclosed ICA	107	14	121	40.1%
Cavernous sinus invasion	142	21	163	54.0%

*ACP* adamantinomatous craniopharyngioma, *PCP* squamous papillary craniopharyngioma, *ICA* internal carotid artery

### Model performance and intra-group analysis

**Fig. 4:**
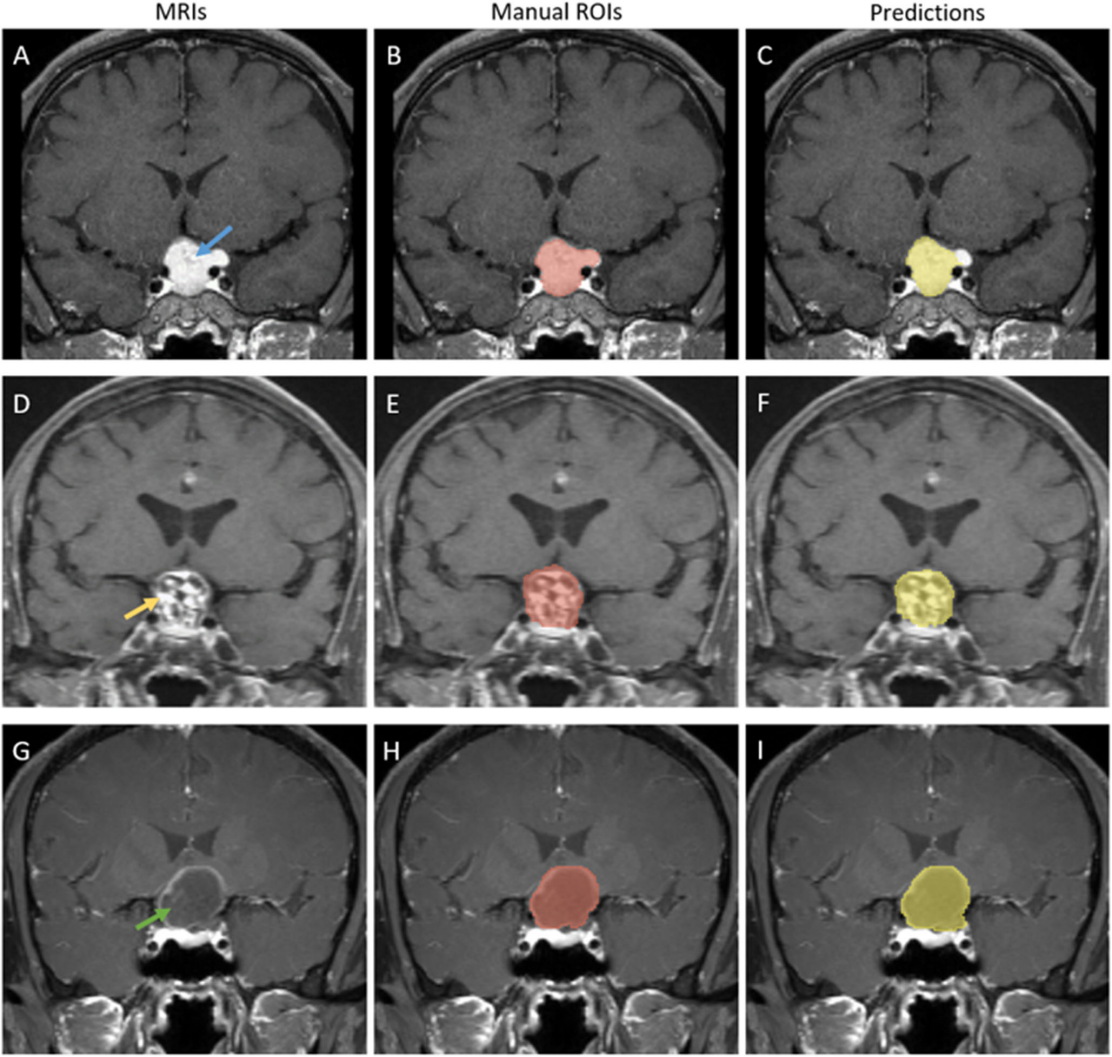
Tumor segmentation results of craniopharyngiomas from center A (internal test). In the labeled images, blue arrow represents solid composition of tumor, green arrow represents darken cyst of tumor. **A**–**C** A case with predominantly solid composition. Segmentation performance of this case is DSCs of 0.865, 95HD of 4.975 mm. **D**–**F** A case with predominantly mixed solid-cystic composition. Segmentation performance of this case is DSCs of 0.858, 95HD of 2.236 mm. **G**–**I** A case with predominantly cystic composition. Segmentation performance of this case is DSCs of 0.791, 95HD of 6.001 mm

As shown in Table 2, classic U-net model showed limited performance in tumor segmentation, with average DSCs of 0.767, Jaccard of 0.647, TPR of 0.802, FPR of 0.001, and 95HD of 5.726 mm. The modified model achieved improved performance in the internal test (center A) with DSCs of 0.840, Jaccard of 0.734, TPR of 0.820, FPR of 0.000, and 95HD of 3.669 mm. More results of ablation experiment regarding to the CNN model modification is provided in Supplement Material 2. Interrater reliability for automated volumetric assessment was good with an ICC of 0.987. The sample of the modified model segmentation from internal test group is shown in Fig. 4. Bland Altman plot indicated that the automated method performed as good as expert manual segmentation, as presented in Supplement Material 3.

**Table 2:** CNN Models’ performance in internal test and external test

		Source	DSCs	Jaccard	TPR	FPR	95HD	Volume ICC
Classic U-Net	Internal test	Center A	0.767 (0.701~0.833)	0.647 (0.573~0.721)	0.802 (0.713~0.890)	0.001 (0.0004~0.0008)	5.726 (3.860~7.591)	0.977 (0.951~0.990)
	External test	Center B	0.754 (0.679~0.829)	0.646 (0.571~0.721)	0.736 (0.651~0.822)	0.000 (0.0004~0.0006)	6.676 (4.695~8.657)	0.954 (0.829~0.982)
Modified U-net	Internal test	Center A	0.840 (0.803~0.877)	0.734 (0.685~0.782)	0.820 (0.770~0.870)	0.000 (0.0002~0.0004)	3.669 (2.534~4.803)	0.987 (0.966~0.994)
	External test	Center B	0.816 (0.773~0.858)	0.704 (0.650~0.761)	0.765 (0.703~0.828)	0.000 (0.0002~0.0004)	4.201 (3.294~5.109)	0.978 (0.957~0.989)

*DSCs* dice similarity coefficients, *95HD* Hausdorff surface distance of 95% percentile, *TPR* true positive rate, *FPR* false positive rate, *ICC* interclass correlation coefficients

Four radiological features were evaluated, including predominantly composition, lobulated shape, compressed or enclosed ICA, and cavernous sinus invasion. The results of one-way ANOVA suggested there was no significant difference within the intra-groups, demonstrating that the model performance was not statistically associated with radiological characteristics of tumors (DSCs: *p* = 0.370, 0.353, 0.809, 0.283, respectively; 95HD: *p* = 0.504, 0.508, 0.113, 0.401, respectively), as provided in Table 3. An overview of metrics (DSCs and 95HD) for segmentation performance obtained using modified CNN model are summarized in box plots in Supplement Material 4 and Supplement Material 5.

**Table 3:** Relationship between modified model performance in segmentation and radiological features of tumors

	DSCs	*p* value	Jaccard	*p* value	TPR	*p* value	FPR	*p* value	95HD	*p* value
Predominantly composition		0.370		0.358		0.501		0.617		0.504
Cystic	0.861		0.763		0.837		0.000		3.213	
Solid	0.873		0.769		0.858		0.000		2.865	
Mixed	0.810		0.696		0.790		0.000		4.317	
Lobulated shape	0.860	0.353	0.759	0.378	0.814	0.830	0.000	0.947	4.105	0.508
Compressed or enclosed ICA	0.849	0.809	0.736	0.936	0.836	0.589	0.000	0.104	4.692	0.113
Cavernous sinus invasion	0.822	0.283	0.709	0.272	0.816	0.844	0.000	0.144	4.107	0.401

*DSCs* dice similarity coefficients, *95HD* Hausdorff surface distance of 95% percentile, *ICA* internal carotid artery

### Model generalization

**Fig. 5:**
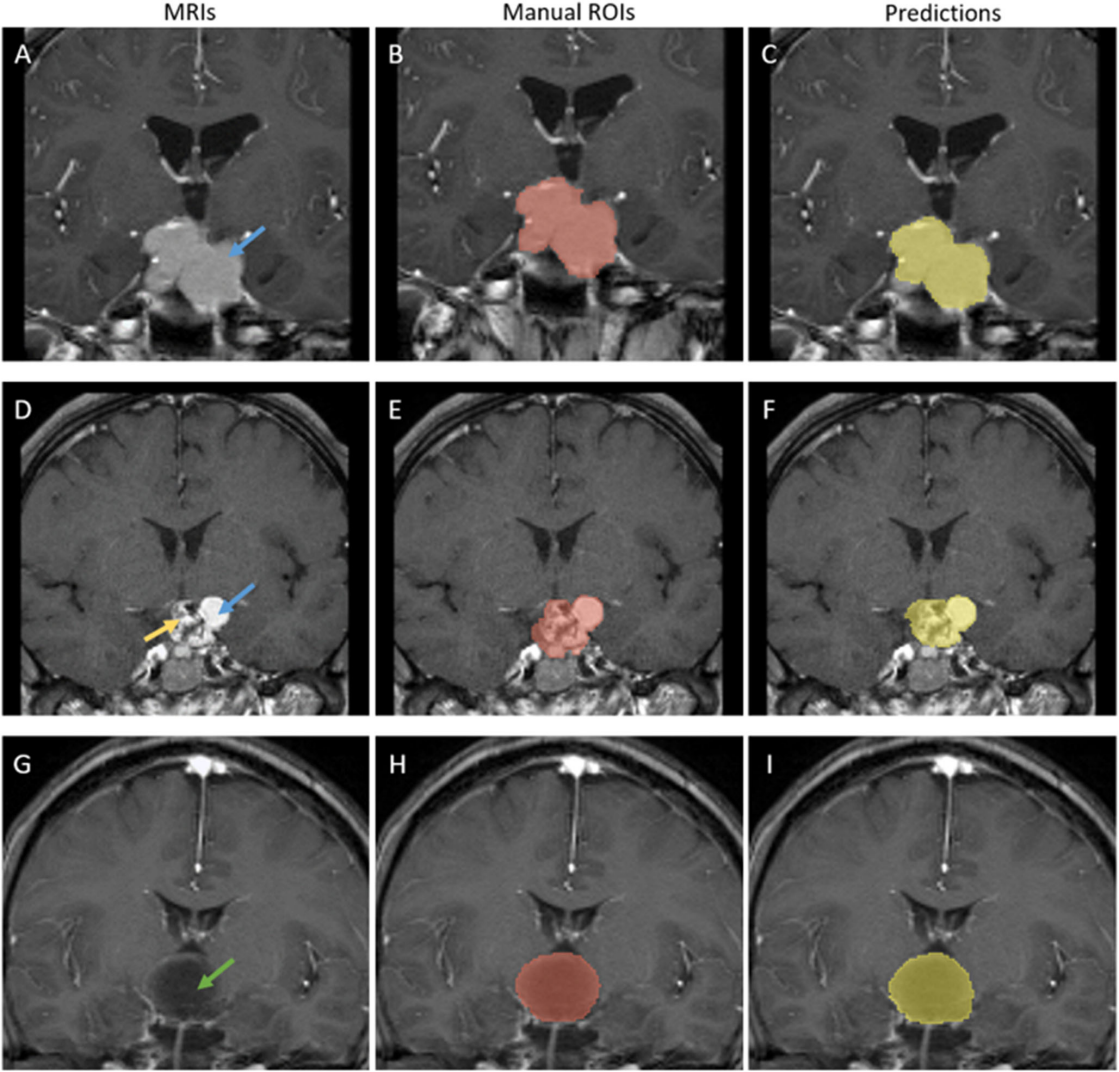
Tumor segmentation results of craniopharyngiomas from center B (independent external test). In the labeled images, blue arrow represents solid composition of tumor, yellow arrow represents mixed composition of tumor, green arrow represents darken cyst of tumor. **A**–**C** A case with predominantly solid composition. Segmentation performance of this case is DSCs of 0.809, 95HD of 3.606 mm. **D**–**F** A case with predominantly mixed solid-cystic composition. Segmentation performance of this case is DSCs of 0.810, 95HD of 4.583 mm. **G**–**I** A case with predominantly cystic composition. Segmentation performance of this case is DSCs of 0.808, 95HD of 6.115 mm

The proposed model also showed to be feasible when tested in the external dataset (center B). The performances were DSCs = 0.816, Jaccard = 0.704, TPR = 0.765, FPR = 0.000, and 95HD = 4.201 mm. The sample of model segmentation from external group is shown in Fig. 5. The performance of the automated method compared to the expert manual segmentation is presented in Supplement Material 6.

### Comparison of radiologists and models

**Fig. 6:**
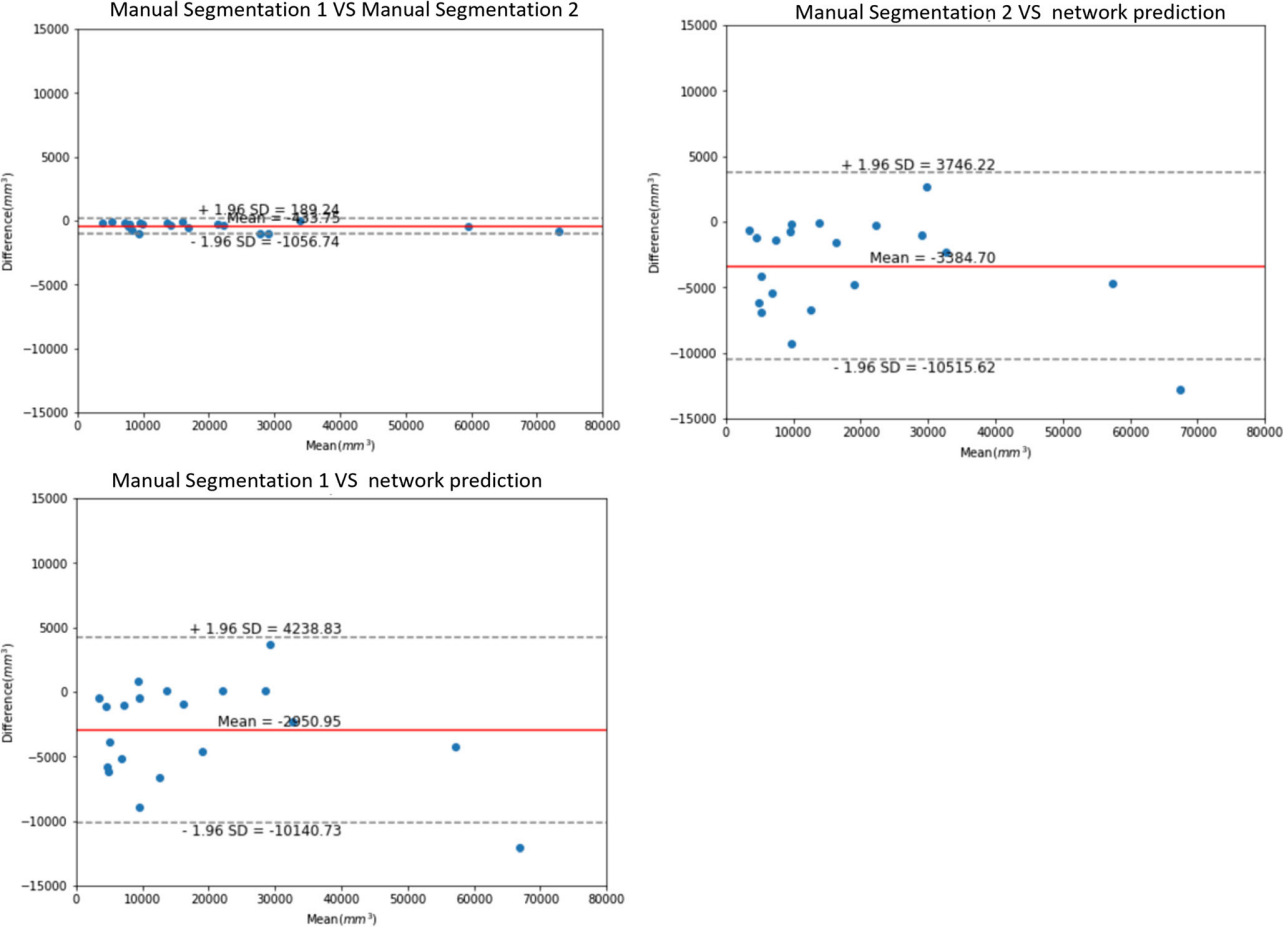
Bland-Altman plots of comparisons among radiologists and model predictions. **A** Intra-observer reproducibility of manual segmentation. **B** 1st time manual segmentation vs model prediction. **C** 2nd time manual segmentation vs model prediction

Averagely, processing time (including image preprocessing and loading time) for tumor segmentation required 3 min 27 s of processing with the CNN model (a laptop equipped with 8-core Intel Core 11800H CPU and NVIDIA 2060 GPU). In contrast, average processing time of radiologists’ segmentation is 23 min 34 s. For the selected 20 cases, Bland-Altman plots suggested that there was good intra-observer reproducibility of manual segmentation. Moreover, it also illustrated that automatic segmentation highly consisted with manual label, as showed in Fig. 6.

## Discussion

In this study, the trained CNN model showed promising results for segmenting craniopharyngiomas with DSCs of 0.840 in the internal test dataset and 0.816 in the external test dataset. Our model significantly outperformed the previous research, demonstrating a new automatic method for craniopharyngioma segmentation [14].

The strong clinical implementation highlights the importance of automated tumor segmentation as it enhances both diagnostic evaluation and tumor growth monitoring. Automated segmentation may also provide an improved approach to routine clinical imaging assessment as it may allow convenient detection, volumetric assessment, and precise therapy planning [25, 26]. Recent advances of radiomics also suggest that automated segmentation can provide an image pre-processing method for subsequent tumor analysis. Comparing to laborious manual delineation, it is highly reproducible and alleviates the inter- and intra-rater variability of clinicians [27].

A lot of the previous researches have attempted to identify methods or algorithms for brain tumor segmentation [28, 29]. Compared to the majority that focused on gliomas and brain metastases, the dramatic variety of image characteristics of craniopharyngiomas sets a higher bar for CNN segmentation [30]. Radiologically, PCP occurs mostly in adults and is predominantly mixed solid-cystic or solid, with spherical shape mass located in suprasellar location. By comparison, ACP generally appears to be a lobulated cystic or predominantly cystic mass located in intrasellar/suprasellar location [31]. Besides, tumor compression or/and invasion of important surrounding tissues, such as hypothalamic and ICA, is also common [1, 32]. Therefore, traditional DNN models could not show promising performances. In the previous research, DNN model developed with classic U-net network showed segmentation ability with DSCs of 0.662, precision of 0.668, and TPR (sensitivity) of 0.677 [14]. Our research also showed similar results, that the performance of U-net was DSCs of 0.767 and TPR of 0.802. When analyzing the results of segmentation, we found that the classic model performed well in segmenting solid entity and cyst walls of tumor, while not feasible in segmenting the darken cyst within the tumor. This result suggested a relatively greater false negative and relatively lower false positive (FP) in prediction. Therefore, we improved this network by adding a non-local attention block to capture long-range dependencies in tumor image, and by setting Tversky index as loss function to improve the sensitivity to false negative pixels, while keeping conservation to false positive pixels. The results suggested that the modification was effective, and our model significantly outperformed the pervious one with DSCs of 0.840. Also, the generality was also showed feasible in the external dataset test, with DSCs of 0.816. However, it should be mentioned that the main purpose of previous research was to build a two-path deep learning system for predicting the invasiveness of craniopharyngioma. The segmentation network they used was only a sub-net in the system. More research regarding deep learning technology is required to validate our results, and to investigate the new network structure for craniopharyngioma segmentation in the future.

One thing should be further discussed was the difference of model performance within the groups. The results showed that there was no significant relationship between model performance and the radiological characteristics, including tumor composition, lobulated shape, compressed or enclosed ICA, and cavernous sinus invasion. Therefore, the provided tumor segmentation algorithm appeared to be feasible for different craniopharyngiomas entities. However, it should be mentioned that compared with homogeneous tumor, the worst results were commonly found in the heterogeneous tumor with mixed solid-cystic composition (Supplement Material 7). Although this finding was not statistically significant, it should be considered because neuroradiologists, neurosurgeons, and neuro-oncologists may need the most assistance from a CNN for lesion volumetric detection. In these cases, manual corrections are still needed to ensure a precise delineation, where the segmentation model presents unsatisfactory performance.

Our research also provided a convenient segmentation tool for subsequent tumor analysis to save some manual efforts. However, we used coronal images for DNN segmentation, while previous researchers usually used axial images for analysis [10, 12, 15, 33]. Coronal images were chosen as it was hard to determine the boundary between the craniopharyngiomas and the invaded cavernous sinus on axial images in some cases. It was important to consider future CAD research that may need most assistance from a DNN models for lesion segmentation. Thus, additional research may be required to further discussed whether or to what extent might radiomics features extracted from coronal images might impact performance for the machine learning classifiers previously provided.

There were several limitations in the current research. First, this was a multi-center, retrospective study with a relatively small data size. Large-scale, multi-center dataset was required to verify our research. Second, the invaded structures were excluded in the current research. For some cases, it should be considered and manual correction should be performed if the clinicians want to get the entire tumor volume. Third, the CNN segmentation model based on U-Net network was only studied in coronal T1CE images, while multimodal images were not included in the research. Previous researches suggested that multisequence feature sets are superior to single-sequence feature sets in tumor image analysis [34, 35]. Automated tumor segmentation in multisequence images should be investigated in future research. Fourth, the methodological investigation in CNN network designing is still limited. For example, the model may be further improved by adding non-local attention block at the earlier layer of the network or adding more than one non-local attention block. Our method is a balance between computation and model performance. With more advanced platform, more complicated, efficient CNN model could be trained to perform segmentation. More computer-science research is required to investigate if the CNN model could be further improved in segmenting craniopharyngiomas.

## Conclusions

This study proposed an automated segmentation DNN model based on U-Net for craniopharyngioma in MRIs. The proposed model showed feasibility in the segmentation, and was a fully automated diagnosis-aid method that could reduce the workload of radiologists. Also, automated segmentation provided a new algorithm for automated craniopharyngioma segmentation in future CAD research.

## Supplementary Information

ESM 1 (PDF 587 kb)

## Abbreviations

95HD: Hausdorff surface distance of 95% percentile
ACP: Adamantinomatous craniopharyngioma
AI: Artificial intelligence
CAD: Computer-aided diagnosis
CE: Cross entropy
CNN: Convolutional neural networks
DNN: Deep neural network
DSCs: Dice similarity coefficients
FPR: False positive rate
ICA: Internal carotid artery
ICC: Interclass correlation coefficients
MRI: Magnetic resonance imaging
PCAS: Picture archiving and communication system
PCP: Squamous papillary craniopharyngioma
ROIs: Regions of interest
T1CE: Contrast-enhanced T1 images
TPR: True positive rate

## Declarations

### Guarantor

The co-scientific guarantor of this publication are Lei Zhang and Jianguo Xu.

### Statistics and biometry

No complex statistical methods were necessary for this paper.

### Methodology

• retrospective

• experimental

• multicenter study
