# Supplementary material for: Automated segmentation of craniopharyngioma on MR images using U-Net-based deep convolutional neural network
Source: Eur Radiol. 2022 Nov 18;33(4):2665–75. doi: 10.1007/s00330-022-09216-1 (PMC10017618; doi:10.1007/s00330-022-09216-1)

ELECTRONIC SUPPLEMENTARY MATERIAL

Automated segmentation of craniopharyngioma on MR images using U-Net-based deep convolutional neural network

Supplement Material 1: Definition of the segmentation metrics.

1. Dice similarity coefficients (DSCs):

$$\mathbf{DSC} = \frac{\mathbf{2} \, * \, (\mathbf{Manual\ label} \cap \mathbf{prediction})}{\mathbf{Manual\ label} + \mathbf{prediction}}$$

2. Hausdorff distance of 95% percentile (95HD):

$$\mathbf{HD} = \mathbf{max}\,(\mathbf{h}(\mathbf{A}, \mathbf{B}), \mathbf{h}(\mathbf{B}, \mathbf{A}))$$

3. Jaccard value:

$$\mathbf{Jaccard} = \frac{|\mathbf{Manual\ label} \cap \mathbf{prediction}|}{|\mathbf{Manual\ label}| + |\mathbf{prediction}| - |\mathbf{Manual\ label} \cap \mathbf{prediction}|}$$

4. true positive rate (TPR): also know as recall rate. Defined as

$$\mathbf{TPR} = \frac{\mathbf{True\ Positive}(\mathbf{TP})}{\mathbf{True\ Positive}(\mathbf{TP}) + \mathbf{False\ Negative}(\mathbf{FN})}$$

5. false positive rate (FPR):

$$\mathbf{FPR} = \frac{\mathbf{False\ Positive}(\mathbf{FP})}{\mathbf{True\ Negative}(\mathbf{TN}) + \mathbf{False\ Positive}(\mathbf{FP})}$$

Supplement Material 2: Experimental results of CNN model modification

Two tricks were used to improve model performance in the current research.

Tversky loss

Tversky loss is introduced to improve the sensitivity to false negative pixels, while keep conservation to false positive pixels. It is defined as:

$$\textbf{Tversky loss} = \frac{\mathbf{2TP}}{\mathbf{2TP} + \boldsymbol{\alpha}\mathbf{FP} + \boldsymbol{\beta}\mathbf{FN}}$$

Hyperparameters were set as $\boldsymbol{\alpha}$ = 0.1, $\boldsymbol{\beta}$ = 0.9

By using the modified loss to the baseline U-Net, the model showed improved performance with average DSCs of 0.816, Jaccard of 0.698, TPR of 0.788, FPR of 0.000, and 95HD of 4.301mm.

Non-local attention block

Non-local attention block was proposed by Wang in 2018. It is an efficient, simple, and generic component for capturing long-range dependencies in deep neural networks. Specifically, this block performs a non-local operation by computing the response at a position as a weighted sum of the features at all positions in the input feature maps. It can be presented as:

$$\boldsymbol{y}i = \frac{1}{C(\mathrm{x})} \sum_{\forall j} f(\boldsymbol{x}i, \boldsymbol{x}j) g(\boldsymbol{x}j)$$

Here, i presents the index of an output position whose response is to be computed; j presents the index that enumerates all possible positions; x presents the input signal (features maps); y presents the output signal of the same size as x. A pairwise function *f* calculates a scalar between i and all j. The unary function *g* calculates a representation of the input signal at the position j. The response is normalized by a factor *C*(x).

To reduce the computational burden of model training, we added this block at the bottom of the network. By adding the non-local block to the baseline U-Net, the model performance in the internal test group was average DSCs of 0.783, Jaccard of 0.667, TPR of 0.807, FPR of 0.001, and 95HD of 4.665mm. The results were briefly summarized as follows:

CNN Models	DCSs	Jaccard	TPR	FPR	95HD
base line U-Net	0.767	0.647	0.802	0.001	5.726 mm
base line U-Net with modified loss	0.816	0.698	0.788	0.000	4.301 mm
base line U-Net with non-local attention block	0.783	0.667	0.807	0.001	4.665 mm
Modified U-net	0.840	0.734	0.820	0.000	3.669 mm

All experiments were performed in a machine equipped with NVIDIA Tesla K40 accelerator (with 12GB RAM, produced by NVIDA corporation, Santa Clara, USA).

Supplement Material 3: Bland-Altman plot for difference in prediction for tumor compartments in center A (internal test). The mean is indicated by the solid line, and 95% confidence intervals are indicated by the dashed lines.

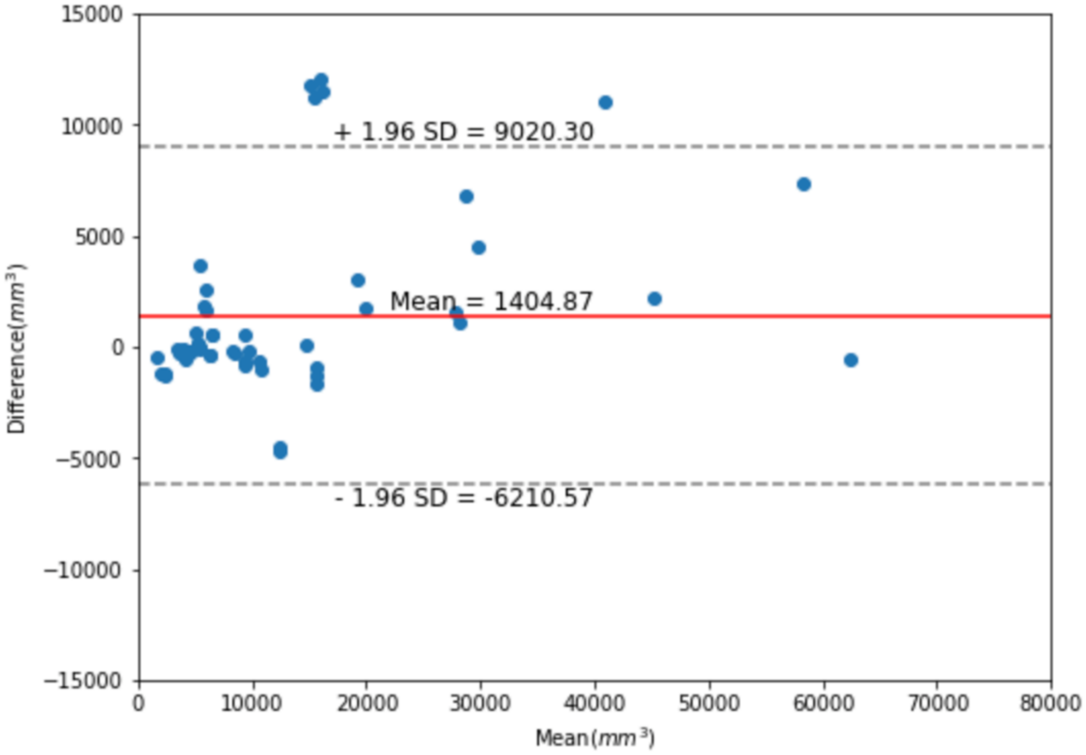

Supplement Material 4: Evaluation results (DSCs) for the modified U-net regarding radiological characteristics of craniopharyngiomas. Blue boxes for predominantly composition, purple boxes for lobulated shape, orange boxes for tumors with or without compressed or enclosed ICA, and green boxes for tumors with or without cavernous sinus invasion.

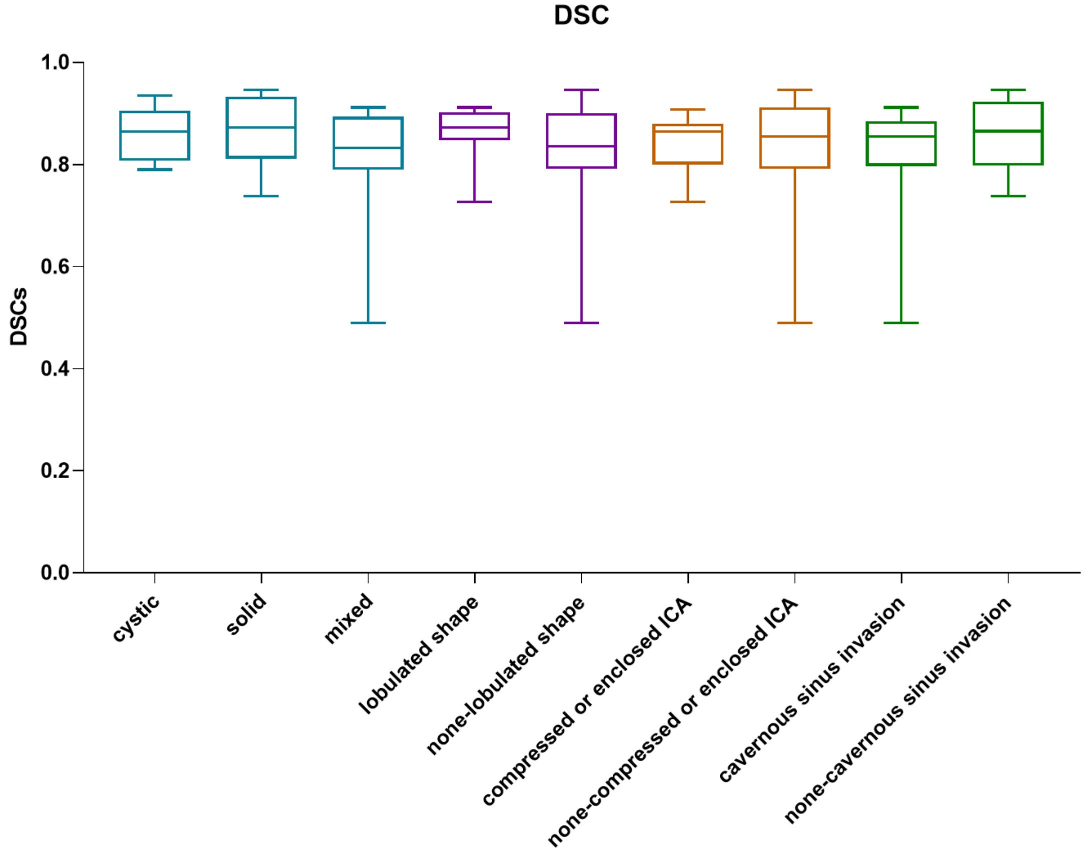

Supplement Material 5: Evaluation results (95HD) for the modified U-net regarding radiological characteristics of craniopharyngiomas. Blue boxes for predominantly composition, purple boxes for lobulated shape, orange boxes for tumors with or without compressed or enclosed ICA, and green boxes for tumors with or without cavernous sinus invasion.

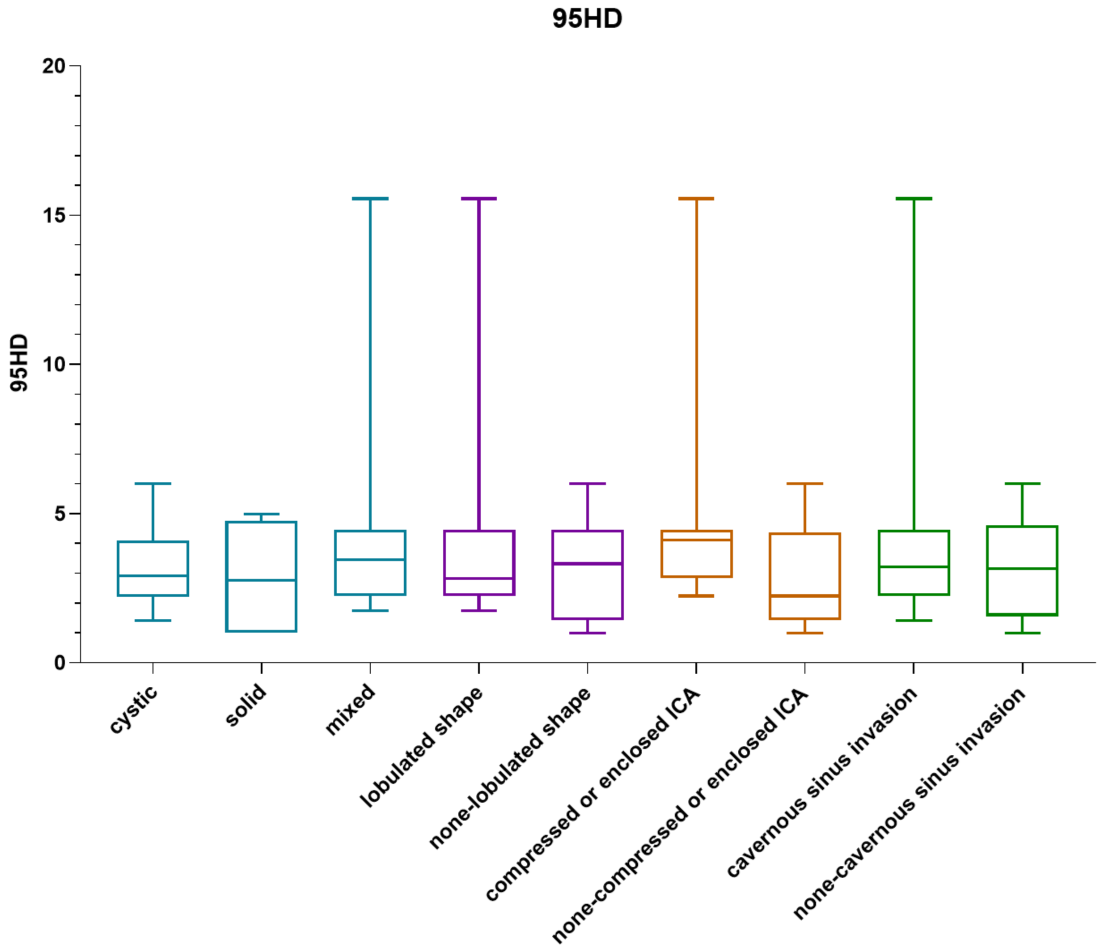

Supplement Material 6: Bland-Altman plot for difference in prediction for tumor compartments in center B (independent external test). The mean is indicated by the solid line, and 95% confidence intervals are indicated by the dashed lines

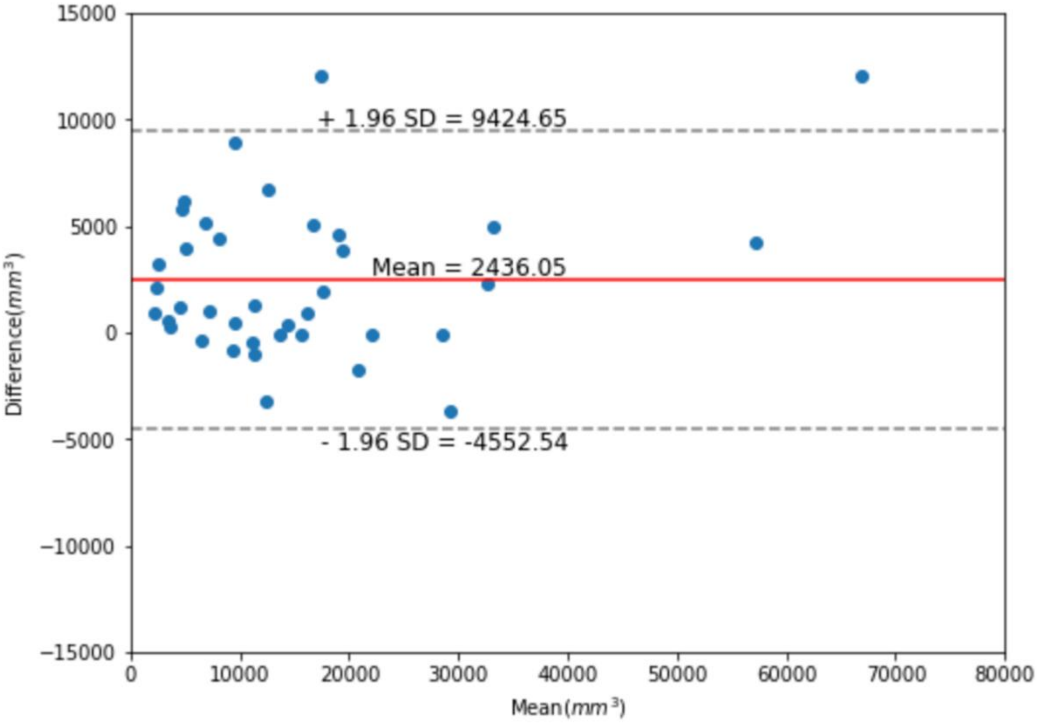

Supplement Material 7: Two cases that the model did not perform well with DSCs lower than 0.700. A)~C) Case 1. DSCs of 0.783, 95HD of 5.775. D)~F) Case 2. DSCs of 0.490, 95HD of 6.431.

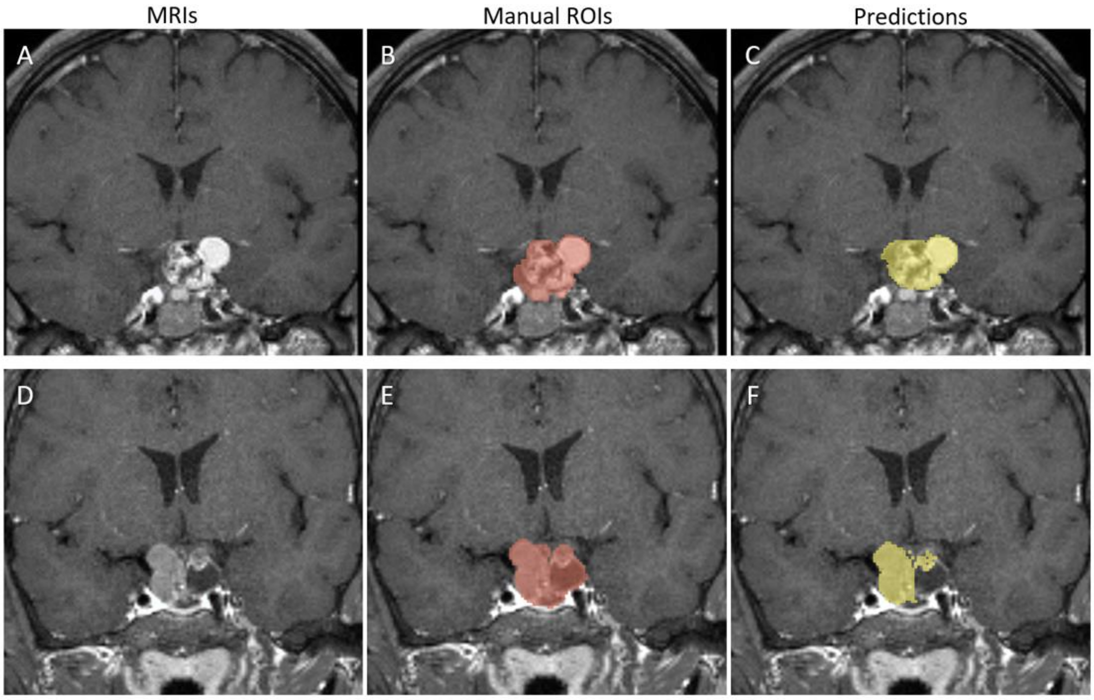

Supplement: Supplementary file 1 — (PDF 587 kb) [file 330_2022_9216_MOESM1_ESM.pdf]
